# Chitosan enhances rosmarinic acid production in shoot cultures of *Melissa officinalis* L. through the induction of methyl jasmonate

**DOI:** 10.1186/s40529-019-0274-x

**Published:** 2019-10-17

**Authors:** Ghazaleh Fooladi vanda, Leila Shabani, Roya Razavizadeh

**Affiliations:** 1 Department of Biology, Faculty of Sciences, Shahrekord University, Shahrekord, Iran; 2 Department of Biology, Payame Noor University, 19395-3697, Tehran, Iran

**Keywords:** Chitosan, Lipoxygenase, Methyl jasmonate, *Melissa officinalis*, Rosmarinic acid

## Abstract

**Background:**

Chitosan is a polycationic polysaccharide derived from chitin that has been recognized as an effective elicitor in the production of secondary metabolites of many medicinal plants. In this study, the effect of abiotic elicitor (chitosan) at various concentrations on rosmarinic acid (RA) and total phenolic accumulation in shoot cultures of lemon balm was investigated.

**Results:**

Treatment of shoots by chitosan led to a noticeable induction of phenylalanine ammonia-lyase (PAL), catalase (CAT), guaiacol peroxidase (GPX) and lipoxygenase (LOX) activities. Besides, the expression of *PAL1*, *TAT* and *RAS* genes and accumulation of RA and phenolic compound increased in chitosan-treated lemon balm shoots. Chitosan treatment also increased H_2_O_2_ accumulation and the expression of *RBOH*, an essential gene implicated in ROS production. Also, the up-regulation of the *OPR* gene by exogenous chitosan was associated with the induction of endogenous JA determined by GC-MASS.

**Conclusion:**

The present study showed that the induced production of rosmarinic acid by chitosan involves the trigger of defense-related enzymes, up-regulated expression of *TAT* and *RAS* genes, and stimulation of JA biosynthesis.

## Background

Rosmarinic acid (RA) biosynthesis was firstly studied in *Mentha x piperita* validating the participation of two parallel pathways for building rosmarinic acid (Ellis and Towers 1970). RA is synthesized by condensation of caffeic acid with 3,4-dihydroxyphenyllactic (Mizukami and Ellis 1991), which the their metabolic origins were found to be l-phenylalanine and l-tyrosine, respectively. l-phenylalanine is transformed to caffeic acid through the phenylpropanoid pathway, which initial reaction in this pathway is catalyzed by phenylalanine ammonia-lyase (PAL). On the other hand, tyrosine aminotransferase (TAT) catalyzes the initial step of the tyrosine-derived pathway leading to a moiety of 3,4-dihydroxyphenyl-lactic acid. PAL and TAT play an important role in the formation of rosmarinic acid (De-Eknamkul and Ellis 1987). Another crucial step in the RA biosynthetic route comprises of transesterification reaction of 4-hydroxyphenyllactate with coumaroyl-CoA catalyzed by rosmarinic acid synthase (RAS). RA compound displays antioxidant and anti-inflammatory effects, as well as antimicrobial activities (Bulgakov et al. 2012).

Chitosan is a deacetylated derivative of chitin and a linear polysaccharide composed of randomly distributed β-(1 → 4)-linked D-glucosamine and N-acetyl-D-glucosamine. In numerous studies, chitosan has improved the yield of many useful compounds, especially total phenolic and rosmarinic acid in vitro culture systems (Chang et al. 1998; Chakraborty et al. 2009; Esmaeilzadeh et al. 2012). Although the exact mechanism of chitosan action in plants is still unknown, several assumptions have been suggested. The responses elicited by chitosan include hydrogen peroxide (H_2_O_2_) production, increased transcription/translation of plant defense genes and phytoalexins (Loschke et al. 1983; Hadwiger 1999). Production of H_2_O_2_ in the early phase of plant response to elicitors has been previously reported (Lin et al. 2005). Lin et al. (2005) showed that chitosan could stimulate a variety of defense responses, including production of H_2_O_2_ and increasing PAL activity. NADPH oxidases or specialized respiratory burst oxidase homologues (RBOHs) play a key role in ROS production. The plant’s NADPH oxidases regulate the signaling cascades in response to abiotic stresses (Suzuki et al. 2011). ROSs are involved in intra-and extracellular cell signaling and preserving cell homeostasis (Ali and Alqurainy 2006). H_2_O_2_ can also act as a signal to stimulate the transcription of genes related to the secondary metabolism production. However, higher concentrations of ROS is destructive for the plant tissues and must be detoxified by antioxidant enzymes such as catalase and peroxidase (Mittler 2002; Cao and Jiang 2006).

Other defense responses elicited by chitosan include the liberation of linolenic acid from membranes and its transformation to the signaling molecule jasmonic acid (JA) (Reymond and Farmer 1998; Hadwiger 1999). In less than three decades, jasmonic acid (JA) and methyl jasmonate (MeJA) were known as significant signals in plant responses to biotic and abiotic stress. Jasmonates are originated from linolenic acid within the lipoxygenase pathway. In this pathway, linolenic acid could be converted into cyclopentanone 12-oxophytodienoic acid (12-oxo-PDA). The transformation of cyclopentenone into cyclopentanone is catalyzed by OPDA reductase (OPR), the critical enzyme in the biosynthetic pathway of JA (Hedden and Thomas 2006).

Although plant responses to chitosan have been extensively studied, how the mechanism of chitosan action for induction rosmarinic acid and phenolic compound production has remained unstudied. Therefore, the present study is focused on (1) investigating the effect of different concentrations of chitosan on the antioxidative capacity, activities of enzymes implicated in the plant defense response (PAL, catalase and peroxidase), production of rosmarinic acid and expressions of genes involved in its biosynthesis, and (2) evaluating the lipoxygenase pathway under different concentrations of chitosan in shoot cultures of lemon balm.

## Materials and methods

### Plant material, culture conditions and chitosan treatment

Seeds of lemon balm were obtained from Pakanbazr seed company (Isfahan Province, Isfahan, Iran). The seeds were rinsed 3 times with tap water followed by 70% (v/v) ethanol for 20 s, surface sterilized by immersing in sodium hypochlorite (20% v/v) for 8 min, and then three times rinsed in sterile distilled water. The sterilized seeds were placed on 1/2MS (Murashige and Skoog) medium with sucrose (13 g/l) and 0.7% agar for germination induction. Germination started within 1 week in a growth chamber (Binder, Germany) at 25 ± 1 °C with a photoperiod of 16 h light under a light intensity of 4000 lx. Two months’ plantlets were used for the experiment. Two grams of shoots, each with 3 nodes, were cultured in 250 ml flasks containing liquid 1/2MS medium supplemented with various concentrations of chitosan. The media were shaken on an orbital shaker (110 rpm at 25 °C, with a photoperiod of 16 h light under a light intensity of 4000 lux). Stock solution of medium molecular weight chitosan (Mw = 190 kDa, viscosity = 200–800 cps and 75–85% deacetylation, Sigma-Aldrich, #448877) was prepared by adding 1 N HCl (5%) dropwise at 60 °C in 15 min (on magnetic stirrer), then diluted with distilled water, adjusted to pH 5.5 (with 1 N, NaOH) and sterilized by autoclaving. Various concentrations of chitosan, including 50, 100 and 150 mg/l were added to the culture medium. The shoots were harvested 7 and 14 days after chitosan treatment. Gene expression analysis, LOX activity, H_2_O_2_, MDA and methyl jasmonate content were performed on day 7. Shoot fresh weight, phenolic and rosmarinc acid content, and PAL activity enzyme was recorded 2 weeks after elicitation.

### H_2_O_2_ Assay

To determine the H_2_O_2_ concentration according to Alexieva et al. (2001), 100 mg of fresh leaves were homogenized in 1.5 ml of TCA (trichloroacetic acid) (0.1%) and centrifuged at 15,000×*g* for 15 min at 4 °C. Then, 500 µl of the supernatant was added to 500 µl of potassium phosphate buffer (10 mM, pH 7.0) and 1 ml of 1 M potassium iodide. After 1 h incubating in the darkness, the absorbance of the mixture was read at 390 nm. Hydrogen peroxide contents of fresh leaves were calculated using a standard curve obtained by the standards of H_2_O_2_.

### Measurement of malondialdehyde

To investigate the membrane damage, the level of malondialdehyde (MDA) was determined in the leaf tissues using the thiobarbituric acid (TBA) test (Hodges et al. 1999). 100 mg of leaf materials were ground in 1.5 ml of 0.1% w/v of trichloroacetic acid (TCA). After 10-min centrifugation at 10,000×*g*, 1 ml of supernatant was added to the same volume of 0.5% w/v of TBA in 20% TCA solution. The sample was mixed vigorously and maintained at 65^◦^C for 30 min. The reaction was discontinued by placing the tubes on ice for 30 min. After 5 min centrifugation at 10,000×*g,* the optical density of the supernatant was detected at 532 nm.

### Total phenolic content

To assay the total soluble phenolic compounds according to the Folin–Ciocalteu method (Singleton and Rossi 1965), 100 mg of leaf samples were ground with methanol (80%) in 1.5 ml tubes. After a 15-min centrifugation at 12,000×*g* at 4 °C, 30 μl of supernatant was added to a volume of 1.5 ml of the assay mixture containing distilled water (470 μl), 2% Na_2_CO_3_ (975 μl) and Folin–Ciocalteu reagent (25 μl). Then samples were then kept at 45 °C for 1 h, and the absorbance was measured at 765 nm. Total phenolic contents of leaf samples were assessed using a standard curve achieved with standards of gallic acid (Sigma-Aldrich, Germany). The contents were stated as μg per 1 g of fresh weight.

### Determination of rosmarinic acid

Rosmarinic acid extraction and analysis followed the methods as described by Ozturk et al. (2010). Two hundred microliter of methanol extracts solutions of shoots were added to 200 µl zirconium oxide chloride solution (0.5 M) and 4.6 ml ethanol. After 5 min, the absorbance of the reaction mixture was measured at 362 nm using a spectrophotometer with rosmarinic acid (Sigma) at the concentration range of 0–0.004 mM as a standard. Rosmarinic acid content in the extracts was expressed as mmol per g fresh weight.

### PAL activity analysis

PAL activity was assayed according to Morrison et al. (1994). The reaction mixture contained 2.5 ml of 0.1 M Tris–HCl (pH 8.5) with 12 mM phenylalanine and 0.5 ml enzyme extract. The reaction mixture without phenylalanine was added as control to each of enzyme extracts. At the onset of reaction and an hour later, an increase in absorbance due to PAL activity was recorded spectrophotometrically at 290 nm. Results were expressed as unit/g fresh weight. One unit of PAL activity is equal to 1 µmol of cinnamic acid produced per 1 min.

### Antioxidant enzymes activities assays

In order to assay the activity of catalase according to the method of Aebi (1984), 100 mg of fresh leaves were homogenized in 1.5 ml of 50 mM phosphate buffer pH of 7.8 containing 0.1 mM EDTA. A 20-min cool centrifugation (at 4 °C) was then performed for the mixture at 12,000×*g*, and then, 500 µl of the supernatant was added to 1.5 ml of sodium phosphate buffer containing 10 mM H_2_O_2_. The change in the optical density (ΔOD) of the solution was measured spectrophotometrically at 240 nm, and CAT activity was reported as unit per 1 g of fresh weight of the plant leaf material. Unit was defined as μmol H_2_O_2_ decomposed per 1 min at 25 °C.

The activity of guaiacol peroxidase was assayed according to Lin and Kao (1999) following the formation of tetraguaiacol. 100 mg of leaf material were homogenized in 1.5 ml of 50 mM phosphate buffer pH 7.8 comprising 0.1 mM EDTA. After a 20-min centrifugation at 12,000×*g* at 4 °C, 50 μl of the supernatant was added to a reaction mixture composed of 50 mM phosphate buffer comprising 19 mM H_2_O_2_ and 9 mM guaiacol. The change in the optical density (ΔOD) of the solution was measured spectrophotometrically at 470 nm, and GPX activity was reported as unit per 1 g of fresh weight of the plant leaf material. One unit of peroxidase was defined as the amount of enzyme that caused the formation of 1 mM of tetraguaiacol per 1 min at 25 °C.

### Lipoxygenase (LOX) activity

Lipoxygenase (LOX) activity was examined as explained by Axelrod et al. (1981). A 100 mg of the leave samples were homogenized in 1.5 ml of 0.1 M Tris–HCl buffer (pH 8.5) comprising 1% PVP (w/v), 1 mM CaCl_2_, and 10% (v/v) glycerol, and centrifuged at 11,000×*g* for 20 min at 4 °C. 50 μl of the supernatant was mixed to a reaction solution consisting of 1.95 ml of 50 mM phosphate buffer (pH 7.0) containing 50 mM linoleate. LOX activity was assayed by observing the alteration in the OD at 234 nm per minute at 25 °C and indicated as ΔOD per 1 g of fresh leaves in 1 min.

### Extraction and analysis of methyl jasmonate by GC-Mass

Methyl jasmonate (MJ) was extracted from the leaves and quantified following the methods reported by Alami et al. (1999). Fresh leaves were extracted with dimethylformamide (DMF) using a pestle and mortar in ice. The liquid extract was separated from the homogenate by centrifuging at 12,000 × *g* for 20 min at 4 °C and analyzed by gas chromatography (Agilent, 5975C) on a fused silica capillary column HP5890GC (30 * 0.25 mm id and 0.25 µm film thickness). After 0.5 min hold at 95 °C, the column temperature shifted to 240 °C at 4 °C/min, and the temperature of injector and detector were set at 240 °C and 250 °C, respectively. Hydrogen was used as the mobile phase, flowing at a rate of 1 ml. min^−1^ . MeJA was identified according to a MeJA standard (Sigma-Aldrich) retention time, and quantified using a standard curve prepared with known concentrations of MeJA.

### Quantitative real-time PCR assay

The extraction of total RNA from shoots and the synthesis of cDNA were performed as described earlier (Mousavi and Shabani 2019). QPCR for β-actin (housekeeping gene), *RAS, PAL1, TAT, OPR3* and *RBOH* cDNAs were carried out using the Qiagen apparatus (Qiagen Rotor-Gene, CA, USA). Reaction mixture contained SYBR Premix Ex Taq (TaKaRa, cat. no. RR081Q), specific primer pairs (Table 1) and cDNA template. The sequences of the rosmarinic acid synthase (RAS) and PAL genes were obtained from the NCBI database (GenBank accession no. FR670523.1 and FN665700.1, respectively). The primers for beta-actin genes (Actin) and tyrosine aminotransferase (TAT) were taken from Doring et al. (2014). RNA-seq data (GSE100970) was used for design primers of *RBOH* gene (NADPH oxidase). The thermocycler program for all reactions was set at 95 °C for 3 min; 40 amplification cycles of 95 °C for 20 s, 57 °C for 30 s and 72 °C for 30 s. At the end of PCR, to evaluate specific amplification of the target genes, melting curves ranging from 60 to 95 °C were also included in each run. The details of amplification efficiency and correlation coefficient (*R*^2^) of the primer pair are given in Table 1. For each selected genes three biological replicates were assayed independently and the threshold cycle (Ct) determined in triplicate. The relative expression levels were analyzed using the 2^−ΔΔCt^ method so that, the differences in Ct value between an unknown sample and the calibrator are reported as fold-changes relative to the calibrator sample.

**Table 1:** Sequences of primers used in this study

Gene name	Primer sequence (5′–3′)	Product length	Efficiency (%)	PCR correlation coefficient (R^2^)
*β*-*Actin*	Actin-f TGTATGTTGCCATCCAGGCCG Actin-r AGCATGGGGAAGCGCATAACC	0.996	98.2	128
*RAS*	RAS-f ACGCCCCGACCTCAACCTTATC RAS-r AAGTGGTGCTCGTTTGCCACG	0.991	97.4	128
*PAL1*	PAL-f GCCGAAGTCATGAACGGAAAGC PAL-r CGCAGCCTTAACATAACCGCTC	0.996	96.8	128
*TAT*	TAT-f CCTACAAGCTACCAGCCGACTC TAT-r AGCCCGTAGATTGGGAAACACG	0.993	96.1	120
*OPR3*	OPR3-f TGCGGGAACCCTGCGGAATA OPR3-r ATGCAGAGCGCTGAACGCCA	0.991	96.5	126
*RBOH*	RBOH-f ATTGGAGCAATGGCGGCGCT RBOH-r AGTGTTGCGGCACATCGGCA	0.996	96.3	159

### Data analysis

The data were analyzed by ANOVA in the computer software statistical analysis system (SAS version 8). All experimental data show as the mean and standard deviation of triplicate experiments. Treatment mean comparisons were performed with the least significant difference (LSD).

## Results

**Fig. 1:**
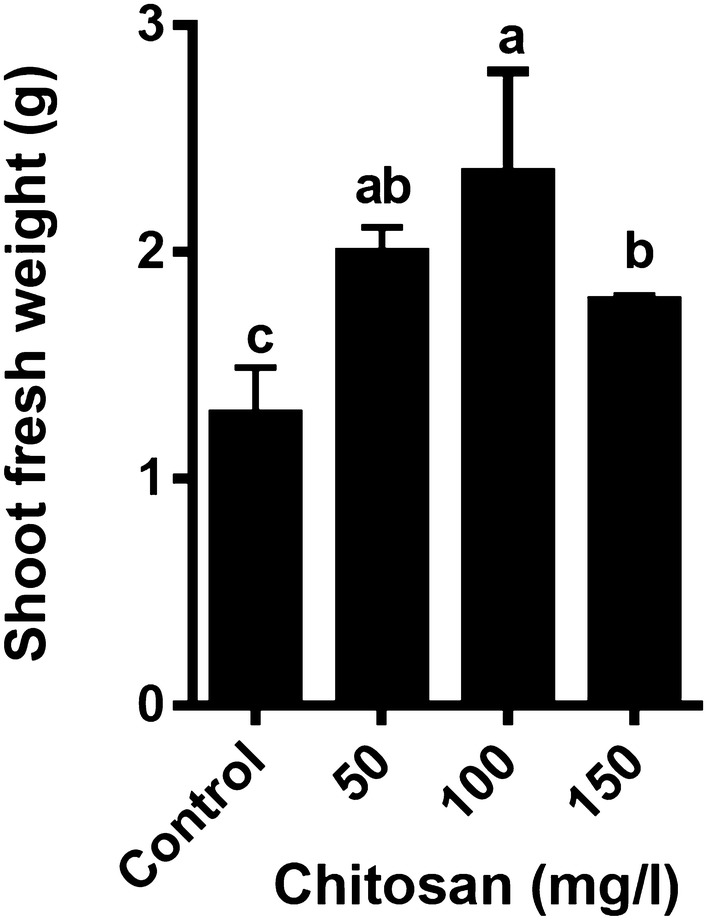
Effect of chitosan levels on SFW of *Melissa officinalis* shoot cultures. Each value is expressed as mean ± standard deviation. Differences were significant at p < 0.05 level (one-way ANOVA followed by LSD test)

**Fig. 2:**
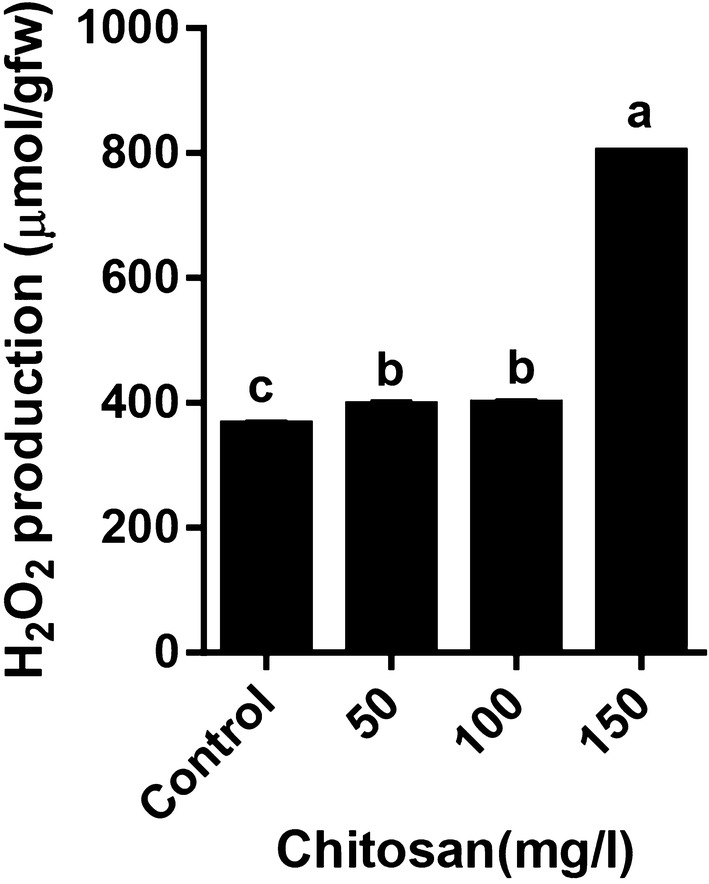
Effect of chitosan levels on H_2_O_2_ content of *Melissa officinalis* shoot cultures. Each value is expressed as mean ± standard deviation. Differences were significant at p < 0.05 level (one-way ANOVA followed by LSD test)

We studied the fresh weight of shoot cultures of lemon balm within 2 weeks. The application of exogenous chitosan resulted in a significant increase of shoot fresh weight (SFW). The results revealed that the highest SFW was seen in 100 mg/l chitosan compared to the control (Fig. 1). Treatment of the shoots of *M. officinalis* with 150 mg/l chitosan led to increases H_2_O_2_ production up to twofold, compared to control (Fig. 2). Other concentrations of chitosan 50 and 100 mg/l did not significantly change the levels of H_2_O_2_ production. An increase in MDA content was observed in all concentrations of chitosan compared to control, but this increase was not significant (data not shown).

**Fig. 3:**
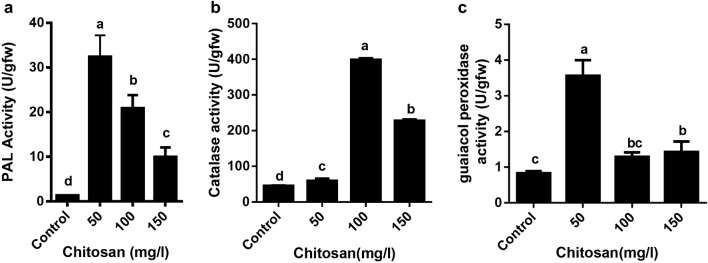
Effect of chitosan levels on PAL (**a**), CAT (**b**) and GOP (**c**) activities of *Melissa officinalis* shoot cultures. Each value is expressed as mean ± standard deviation. Differences were significant at p < 0.05 level (one-way ANOVA followed by LSD test)

In this study the application of 50 mg/l chitosan resulted in the highest PAL activity (32.46 U/gfw), while the lowest was observed in 150 mg/l chitosan which registered 2.1 U/gfw (Fig. 3a). Activity of the catalase enzyme increased with increasing chitosan concentration, and the activity reached a maximum at 100 mg/l chitosan (Fig. 3b). According to the results presented in the Fig. 3c, treatment of shoots with 50 mg/l chitosan caused increase in guaiacol peroxidase (GPX) activity up to about fourfold higher than control. There were no significant differences between the GPX activity of control plants and plants treated with 100 and 150 mg/l of chitosan.

**Fig. 4:**
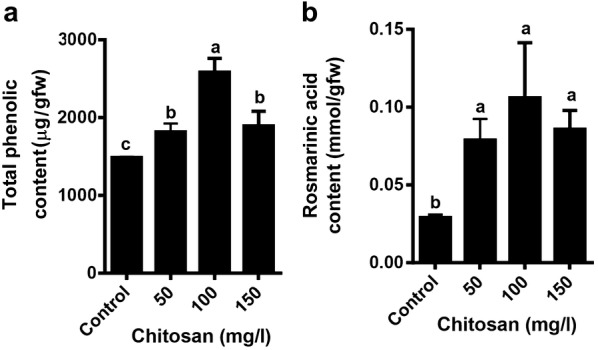
Effect of chitosan levels on total phenolic compound (**a**) and rosmarinic acid (**b**) of *Melissa officinalis* shoot cultures. Each value is expressed as mean ± standard deviation. Differences were significant at p < 0.05 level (one-way ANOVA followed by LSD test)

An increase in the production of total phenolic compounds was observed in the all concentrations of chitosan compared to control. The results revealed that the highest phenolic compound content was found in 100 mg/l chitosan (Fig. 4a). Also, rosmarinic acid content was increased in all concentrations of chitosan without any significant differences (p < 0.05) between treatments. Rosmarinic acid content registered a threefold after 2 weeks when compared to the control (Fig. 4b).

**Fig. 5:**
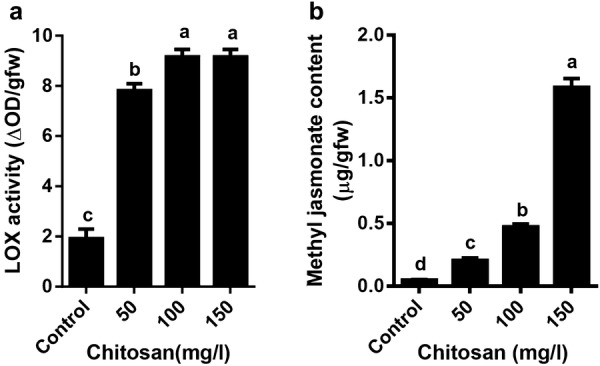
Effect of chitosan levels on lipoxygenase activity (**a**) and methyl jasmonate content (**b**) of *Melissa officinalis* shoot cultures. Each value is expressed as mean ± standard deviation. Differences were significant at p < 0.05 level (one-way ANOVA followed by LSD test)

Figure 5 shows the effect of chitosan on LOX activity and endogenous methyl jasmonate (MJ) content. Chitosan significantly increased LOX enzyme activity at all concentrations compared to control. However, there was no significant difference between 100 and 150 mg/l chitosan (Fig. 5a). Chitosan at 150 mg/l gave the highest amount of MJ (1.58 ± 0.06 µg/gfw), which was 32-fold higher than the control at 1 week after elicitation (Fig. 5b).

**Fig. 6:**
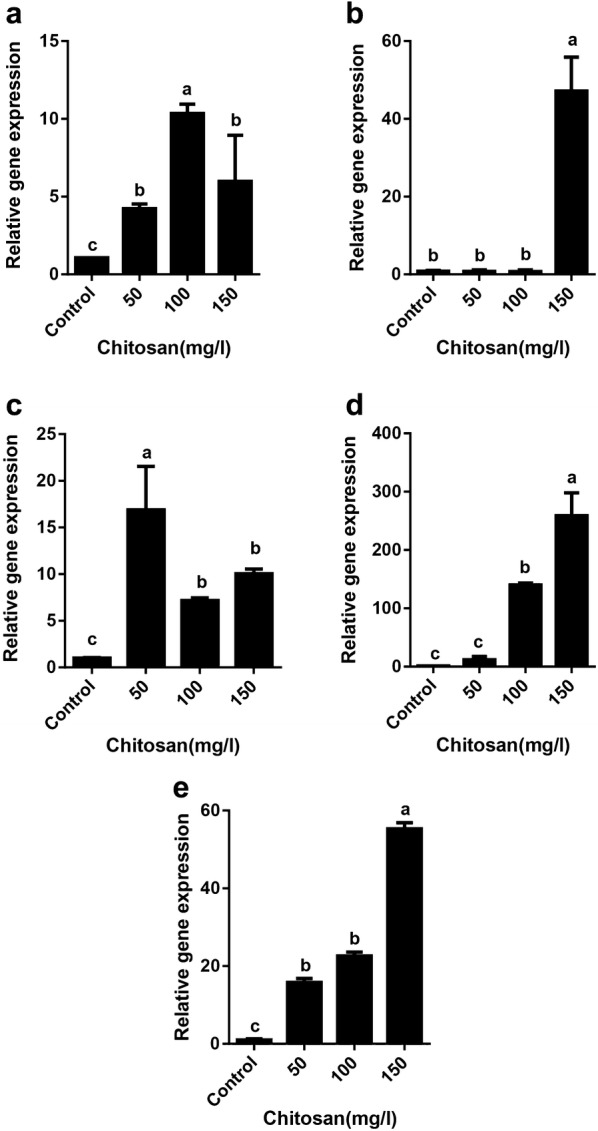
Effect of chitosan levels on gene expression of *TAT* (**a**), *PAL* (**b**), *RAS* (**c**), *RBOH* (**d**) and *OPR3* (**e**) of *Melissa officinalis* shoot cultures. Each value is expressed as mean ± standard deviation. Differences were significant at p < 0.05 level (one-way ANOVA followed by LSD test)

The effect of exogenous chitosan on gene expression induction was investigated using real-time PCR analysis (Fig. 6). Evaluation of the gene expression indicated that there was a significant enhancement of *TAT* expression in all concentration of chitosan compared with  the control (Fig. 6a). A significant increment in *PAL1* gene expression (47.17 ± 8.7, p < 0.05) was observed only in 150 mg/l of chitosan (Fig. 6b). For *RAS* gene, the exogenous chitosan triggered an increase of *RAS* transcripts at all concentrations and the highest expression was found in 50 mg/l chitosan compared to the control (Fig. 6c). The expression of *RBOH* was significantly (p < 0.05) up regulated in 100 mg/l and 150 mg/l chitosan compared with the control (Fig. 6d). The expression of *OPR3* showed a significant enhancement in the three concentrations of chitosan (15.82 ± 1.22, 22.62 ± 2.42 and 55.34 ± 1.53, respectively, p < 0.05). There was no significant difference in *OPR3* expression between 50 and 100 mg/l chitosan (Fig. 6e).

## Discussion

Several studies were performed on the biotechnological production of RA to obtain high yields of this hydroxylated phenolic compound. However, the impact of different elicitors, including methyl jasmonate, salicylic acid, abscisic acid, yeast elicitor, silver ions and chitosan on the phenolic compounds and rosmarinic acid production has been shown in some studies (Chen and Chen 2000; Bais et al. 2002; Yan et al. 2006; Dong et al. 2010; Wang et al. 2011; Hao et al. 2012). Although plant responses to elicitors have been extensively studied, how to increase rosmarinic production under chitosan treatment has remained unstudied.

The metabolic changes induced by elicitation may be part of the mechanisms of adaptation to biotic or abiotic elicitors or otherwise including mechanisms of damage caused by exposure to stress conditions in the plants. They may therefore result in growth stimulation or growth limitation. In the present study, the highest shoot biomass was observed in 100 mg/l chitosan compared to the control. Numerous studies indicated that chitosan has a potential to enhance the plant growth and yield (Chibu et al. 2002; Khan et al. 2002; Chandrkrachang et al. 2005; Gornik et al. 2008; Algam et al. 2010; Sathiyabama et al. 2016). These effects have been attributed to such criteria as the increased uptake of water and essential nutrients, cell division and elongation, increased protein biosynthesis (Amin et al. 2007) and induction of the antioxidant defense system (Agrawal et al. 2002; Ma et al. 2014). Moreover, the enhancement of rosmarinic acid and phenolic compound production under chitosan treatment was coordinated with the growth rate of shoots. The increase in water uptake and production of secondary metabolites may explain the significantly greater biomass in chitosan-treated shoots.

The results of this study indicated that chitosan treatment increased H_2_O_2_ accumulation (Fig. 2) and also stimulated the expression of *RBOH* gene in shoot cultures of lemon balm. Malerba (2015) have reported increased accumulation of ROS, namely O^2•−^ and H_2_O_2_ in *Acer pseudoplatanus* L. cultured cells as a result of application of chitosan in culture medium. Chitosan induced the activity of CAT enzyme at all concentrations. The activity of GPX significantly increased at low concentration of chitosan (50 mg/l), but did not change at higher concentrations (100 and 150 mg/l). Chitosan stimulates the activities of ROS-scavenging enzymes, including catalase, peroxidase, and polyphenol oxidase, as well as defense-related enzymes, including PAL, chitinase and β-1,3 glucanase (Ma et al. 2014). Apparently, increased H_2_O_2_ accumulation in chitosan treated shoots may have activated these antioxidant enzymes, to counteract the increase in reactive oxygen species (ROS) induced at treatment condition. Despite high H_2_O_2_ accumulation under high concentration of chitosan, it was just CAT but not GPX that showed enhanced enzyme activity. The activity of catalase increased more than fourfold in the 100 mg/l chitosan and twofold in the 150 mg/l chitosan, thus the increased catalase activity appear to compensate the H_2_O_2_ accumulation under high concentrations of chitosan. In this study, no significant increase was observed in activity of GPX enzyme under high concentration of chitosan, which might be caused by the superoxide scavenging ability in high concentration of chitosan.

Similarly, PAL activity was significantly increased by chitosan at all concentrations with the highest value observed in 50 mg/l chitosan (increased by 24-folds, compared to the control). Several investigators reported that chitosan induces the activities of PAL, catalase and peroxidase in plants (Falcon-Rodriguez et al. 2009; Ma et al. 2014; Kuyyogsuy et al. 2018).

The elicitation of shoot cultures by 50, 100 and 150 mg/l chitosan increased the production of total phenolic compound and rosmarinic acid in *M. officinalis*. As shown in Fig. 6, the expressions of *TAT* and *RAS* genes were significantly upregulated at all concentrations of chitosan, but that of *PAL1* gene only increased at 150 mg/l of the elicitor. From the result of total phenolic content and PAL activity, it is quite clear that there is a positive relation between PAL activity and the accumulation of total phenolics as they showed a similar trend. However, the molecular regulation of total phenolic content is related with the expression of *PAL* gene, responsible for playing the pivotal role in total phenolic production through the phenylpropanoid pathway. In many plants, PAL isoforms are encoded by multi-gene families (Wanner et al. 1995). Some of which are only activated in specific tissue or under certain environmental signals. Translation and subsequent protein modification yields the active PAL enzyme, which then stimulates the phenylpropanoid pathway. Gayoso et al. (2010) reported that of the six *PAL* genes examined in tomato roots, *PAL2* was the most highly expressed, followed by *PAL3*, *PAL4*, and *PAL6*. It seems that the effect of chitosan on the level of *PAL1* transcripts is dependent on concentrations of chitosan; maybe the elicitation by low concentrations of chitosan (50 and 100 150 mg/l) induced other isoforms of *PAL* gene in shoot cultures of lemon balm.

Enhancing the enzymes activity and expression of the genes related to RA biosynthesis (*PAL*, *TAT*, and *RAS*) by adding elicitors have been studied slightly in various plants. These elicitors include MJ in cell suspension cultures of *Agastache rugosa* (Kim et al. 2013), yeast extract in *Lithospermum erythrorhizon* cell suspension cultures (Mizukami et al. 1992) and yeast extract, Ag^+^ and MJ in *S. miltiorrhiza* hairy roots (Yan et al. 2006; Zhang et al. 2014). The increase in the antioxidant compounds of shoot cultures of lemon balm by 50 and 100 mg/l chitosan could be due to the stimulating effect of chitosan on the levels of *TAT* and *RAS* transcripts.

However, various reports indicate the role of one of two branching pathways in the biosynthesis of rosmarinic acid. Some of them showed that the biosynthesis of RA and phenolic compounds were correlated with TAT but not PAL activity (for example: Yan et al. 2006; Lu et al. 2013; Zhang et al. 2014; Ru et al. 2017), and the others conversely showed the involvement of PAL-derived pathway (for example: Mizukami et al. 1992; Kim et al. 2013). TAT is presumed to have a role in RA accumulation. For example, Lu et al. (2013) and Kim et al. (2014) emphasized the importance of *TAT* gene in the production of RA in *Perilla frutescens* and *Scutellaria baicalensis* (belonging to the Lamiaceae family), respectively. PAL-derived pathway is more important than TAT-derived pathway in RA biosynthesis of *Dracocephalum tanguticum* (Li et al. 2017). Our results showed that *TAT* gene expression was positively correlated with the production of phenolic compound and RA (R^2^ = 0.96 and R^2^ = 0.87, respectively), highlighting its importance in the regulation of phenolic compound and RA biosynthesis. These results suggest that TAT-derived pathway would be more likely to induce RA accumulation by chitosan in the shoot cultures of lemon balm than PAL-derived pathway.

Our results indicated that 150 mg/l of chitosan induced upregulation of *OPR3* gene, causing the endogenous MJ content to be 32-fold higher than the control. Higher expression of *OPR* genes under several biotic and abiotic stress factors and signaling molecules have been reported (Biesgen and Weiler 1999; Zhang et al. 2005; Li et al. 2011). Yan et al. (2006) found that chitosan could cause upregulation of an important jasmonic acid (JA) synthase (*OPR*) gene in *Brassica napus*. Our results suggest that *OPR3* was critical for MJ biosynthesis in the shoot cultures of lemon balm. During 1-week of elicitation with chitosan, the activity of lipoxygenase (LOX) activity increased 4 times more than the untreated control. In plants, fungi, algae and mammals, LOX are implicated in the production of important signaling molecules and defense metabolites (Andreou et al. 2009). Numerous studies have reported that the elicitors have a potential to enhance the activity of LOX enzyme in plants (e.g. yeast extract in *Cupressus lusitanica* cell culture (Zaho and Sakai 2003); chitosan in grapevine leaves (Trotel-Aziz et al. 2006); and diosgenin in hairy root cultures of *Trigonella foenum*-*graecum* (Merkli et al. 1997), curcumin production in turmeric (Sathiyabama et al. 2016). In some plant cell cultures, LOX activity and jasmonate accumulation were stimulated in response to the elicitor treatment and seemed to take part in the signal transduction pathway foregoing the activation of defense gene expression and the formation of further secondary metabolite such as flavonoids, alkaloids, terpenoids or coumarins (Rakwal et al. 2002). It is well known that jasmonate induces enzymes involved in phenolic compound synthesis like chalcone synthase (CHS) and PAL (Creelman et al. 1992; Gundlach et al. 1992). Several studies have reported the effect of chitosan on increasing endogenous JA levels (Doares et al. 1995; Rakwal et al. 2002). In the present research, we demonstrate that LOX activity is strongly correlated with MJ production.

Oxylipins (oxidized metabolites of unsaturated fatty acids) are products of two non-enzymatic and enzymatic peroxidation of PUFA in the membrane (Wasternack 2007). In the current study, both non-enzymatic (MDA content) and enzymatic of peroxidation of PUFA were examined. Treatment of lemon balm shoot cultures by chitosan induced LOX activity. However, no significant increase was observed in MDA production 1 week after elicitation. These results suggest that upon the elicitation with chitosan, the enzymatic metabolic processes of peroxidation of PUFA would be more likely to be activated than non-enzymatic processes.

## Conclusion

Taken together, the present results suggest that shoot cultures of lemon balm can benefit from chitosan elicitation for a better growth and better antioxidant properties. The results showed that chitosan elicitor only affected *PAL* gene expression at concentration of 150 mg/l, but increased *TAT* gene expression at all concentrations of chitosan. Therefore, it seems that the application of chitosan in lemon balm did not have much activity in the phenylpropanoid pathway and the tyrosine-derived pathway was able to produce more rosmarinic acid. The favorable effect of chitosan elicitor on increasing rosmarinic acid content in shoot cultures of lemon balm is probably due to changes in lipoxygenase activity and *OPR3* gene upregulation, which was associated with increased endogenous methyl jasmonate. And in turn, it seems that methyl jasmonate as a message delivery molecule plays an important role in the production of hydrogen peroxide, induction of key genes expression involved in the biosynthetic pathway of phenolics (*PAL*, *TAT*, and *RAS*), production of defense proteins such as catalase under chitosan elicitation. Therefore, it can be concluded that some of the responses of lemon balm to chitosan elicitor have been through activation of the octadecanoid biosynthetic pathway.

## Data Availability

The datasets analysed during the current study are available from the corresponding author on reasonable request.

## Abbreviations

CAT: catalase
GPX: guaiacol peroxidase
H_2_O_2_: hydrogen peroxide
LOX: lipoxygenase
MJ: methyl jasmonate
PAL: phenylalanine ammonia-lyase
ROS: reactive oxygen species
RA: rosmarinic acid

## References

[CR1] Aebi H (1984). Catalase in vitro. Methods Enzymol.

[CR2] Agrawal GK, Rakwal R, Tamogami S, Yonekura M, Kubo A, Saji H (2002). Chitosan activates defense/stress response(s) in the leaves of *Oryza sativa* seedlings. Plant Physiol Biochem.

[CR3] Alami I, Jouy N, Clervet A (1999). The lipoxygenase pathway is involved in elicitor-induced phytoalexin accumulation in plane tree (*Platanus acerifolia*) cell-suspension cultures. J Phytopathol.

[CR4] Alexieva V, Sergiev I, Mapelli S, Karanov E (2001). The effect of drought and ultraviolet radiation on growth and stress markers in pea and wheat. Plant Cell Environ.

[CR5] Algam S, Xie G, Li B, Yu S, Su T, Larsen J (2010). Effects of *Paenibacillus* strains and chitosan on plant growth promotion and control of *Ralstonia wilt* in tomato. J. Plant Pathol.

[CR6] Ali AA, Alqurainy F, Motohashi N (2006). Activities of antioxidants in plants under environmental stress. The lutein-prevention and treatment for diseases.

[CR7] Amin AA, Rashad M, El-Abagy HMH (2007). Physiological effect of indole-3-butyric acid and salicylic acid on growth, yield and chemical constituents of onion plants. J Appl Sci Res.

[CR8] Andreou A, Brodhun F, Feussner I (2009). Biosynthesis of oxylipins in non-mammals. Prog Lipid Res.

[CR9] Axelrod B, Cheesbrough TM, Laakso S (1981). Lipoxygenase from soybeans. Methods Enzymol.

[CR10] Bais HP, Walker TS, Schweizer HP, Vivanco JM (2002). Root specific elicitation and antimicrobial activity of rosmarinic acid in hairy root cultures of *Ocimum basilicum*. Plant Physiol Biochem.

[CR11] Biesgen C, Weiler EW (1999). Structure and regulation of OPR1 and OPR2, two closely related genes encoding 12-oxophytodienoic acid-10,11-reductases from *Arabidopsis*. Planta.

[CR12] Bulgakov VP, Inyushkina YV, Fedoreyev SA (2012). Rosmarinic acid and its derivatives: biotechnology and applications. Crit Rev Biotechnol.

[CR13] Cao J, Jiang W (2006). Induction of resistance in Yali pear (*Pyrus bretschneideri* Rehd.) fruit against postharvest diseases by acibenzolar-S-methyl sprays on trees during fruit growth. SciHort.

[CR14] Chakraborty M, Karun A, Mitra A (2009). Accumulation of phenylpropanoid derivatives in chitosan-induced cell suspension culture of *Cocos nucifera*. J Plant Physiol.

[CR15] Chandrkrachang S, Sompongchaikul P, Sangtain S (2005). Profitable spin-off from using chitosan in orchid farming in Thailand. J Metals Mater. Miner.

[CR16] Chang JH, Shin IS, Chung HJ (1998). Improved menthol production from chitosan elicited suspension culture of *Mentha piperita*. Biotechnol Lett.

[CR17] Chen H, Chen F (2000). Effect of yeast elicitor on the secondary metabolism of Ti-transformed *Salvia miltiorrhiza* cell suspension cultures. Plant Cell Rep.

[CR18] Chibu H, Shibayama H, Arima S (2002). Effects of chitosan application on the shoot growth of rice and soybean. Jap J Crop Sci.

[CR19] Creelman RA, Tierey ML, Mullet JE (1992). Jasmonic acid/methyl jasmonate accumulate in wounded soybean hypocotyls and modulate wound gene expression. Proc Natl Acad Sci USA.

[CR20] De-Eknamkul W, Ellis BE (1987). Purification and characterization of tyrosine aminotransferase activities from *Anchusa officinalis* cell cultures. Arch Biochem Biophys.

[CR21] Doares SH, Syrovets T, Weiler EW, Ryan CA (1995). Oligogalacturonides and chitosan activate plant defensive genes through the octadecanoid pathway. Proc Natl Acad Sci USA.

[CR22] Dong J, Wan G, Liang Z (2010). Accumulation of salicylic acid-induced phenolic compounds and raised activities of secondary metabolic and antioxidative enzymes in *Salvia miltiorrhiza* cell culture. J Biotechnol.

[CR23] Doring AS, Pellegrini E, Campanella A, Trivellini A, Gennai C, Petersen M (2014). How sensitive is *Melissa officinalis* to realistic ozone concentrations?. Plant Physiol Bioch.

[CR24] Ellis BE, Towers GH (1970). Biogenesis of rosmarinic acid in *Mentha*. Biochem J.

[CR25] Esmaeilzadeh S, Sharifi M, Safaei N, Behmanesh M (2012). Enhancement of lignan and phenylpropanoid compounds production by chitosan and chitin in *Linum album* cell culture. IJPB.

[CR26] Falcon-Rodriguez AB, Cabrera JC, Ortega E, Martinez-Tellez MA (2009). Concentration and physicochemical properties of chitosan derivatives determine the induction of defence responses in roots and leaves of tobacco (*Nicotiana tabacum*) plants. AJABS.

[CR27] Gayoso C, Pomar F, Novo-Uzal E, Merino F, Ilárduya ÓM (2010). The *Ve*-mediated resistance response of the tomato to *Verticillium dahliae* involves H_2_O_2_, peroxidase and lignins and drives *PAL* gene expression. BMC Plant Biol.

[CR28] Gornik K, Grzesik M, Duda BR (2008). The effect of chitosan on rooting of grape vine cuttings and on subsequent plant growth under drought and temperature stress. J. Fruit Ornam Plant Res.

[CR29] Gundlach H, Miiller MJ, Kutchan TM, Zenk MH (1992). Jasmonic acid is a signal transducer in elicitor-induced plant cell cultures. Proc Natl Acad Sci USA.

[CR30] Hadwiger LA, Jolles P, Muzzarelli RAAA (1999). Host-parasite interactions: elicitation of defense responses in plants with chitosan. Chitin and chitinases.

[CR31] Hao G, Ji HW, Li YL, Shi RJ, Wang JM, Feng L (2012). Exogenous ABA and polyamines enhanced salvianolic acids contents in hairy root cultures of *Salvia miltiorrhiza* Bge. f. alba. Plant OMICS.

[CR32] Hedden P, Thomas SG (2006). Plant hormone signaling.

[CR33] Hodges DM, DeLong JM, Forney CF, Prange RK (1999). Improving the thiobarbituric acid-reactive-substances assay for estimating lipid peroxidation in plant tissues containing anthocyanin and other interfering compounds. Planta.

[CR34] Khan WM, Prithiviraj B, Smith DL (2002). Effect of foliar application of chitin oligosaccharides on photosynthesis of maize and soybean. Photosynthetica.

[CR35] Kim YB, Kim JK, Uddin MR, Xu H, Park WT (2013). Metabolomics analysis and biosynthesis of rosmarinic acid in *Agastache rugose* Kuntze treated with methyl jasmonate. PLoS ONE.

[CR36] Kim YB, Uddina MR, Kim Y, Park CG, Park SU (2014). Molecular cloning and characterization of tyrosine aminotransferase and hydroxyphenylpyruvate reductase, and rosmarinic acid accumulation in *Scutellaria baicalensis*. Nat Prod Commun.

[CR37] Kuyyogsuy A, Deenamo N, Khompatara K, Ekchaweng K, Churngchow N (2018). Chitosan enhances resistance in rubber tree (*Hevea brasiliensis*), through the induction of abscisic acid (ABA). Physiol Mol Plant Pathol.

[CR38] Li W, Zhou F, Liu B, Feng D, He Y, Qi K, Wang H, Wang J (2011). Comparative characterization, expression pattern and function analysis of the 12-oxo phytodienoic acid reductase gene family in rice. Plant Cell Rep.

[CR39] Li H, Fu Y, Sun H, Zhang Y, Lan X (2017). Transcriptomic analyses reveal biosynthetic genes related to rosmarinic acid in *Dracocephalum tanguticum*. Sci Rep.

[CR40] Lin CC, Kao CH (1999). NaCl induced changes in ionically bound peroxidase activity in roots of rice seedlings. Plant Soil.

[CR41] Lin W, Hu X, Zhang W, Rogers WJ, Cai W (2005). Hydrogen peroxide mediates defence responses induced by chitosans of different molecular weights in rice. J Plant Physiol.

[CR42] Loschke DC, Hadwiger LA, Wagoner W (1983). Comparison of mRNA populations coding for phenylalanine ammonia lyase and other peptides from pea tissue treated with biotic and abiotic phytoalexins inducers. Physiol Plant Pathol.

[CR43] Lu X, Hao L, Wang F, Huang C, Wu S (2013). Molecular cloning and overexpression of the tyrosine aminotransferase (TAT) gene leads to increased rosmarinic acid yield in *Perilla frutescens*. Plant Cell Tissue Organ Cult.

[CR44] Ma LJ, Li YY, Yu CM, Wang Y, Li XM, Li N, Chen Q, Bu N (2014). Germination and physiological response of wheat (*Triticum aestivum*) to pre-soaking with oligochitosan. Int J Agric Biol.

[CR45] Malerba M (2015). Cerana R (2015) Reactive oxygen and nitrogen species in defense/stress responses activated by chitosan in sycamore cultured cells. Int J Mol Sci.

[CR46] Merkli A, Christen P, Kapetanidis I (1997). Production of diosgenin by hairy rootcultures of *Trigonella foenum*-*graecum*. Plant Cell Rep.

[CR47] Mittler R (2002). Oxidative stress, antioxidants and stress tolerance. Trends Plant Sci.

[CR48] Mizukami H, Ellis BE (1991). Rosmarinic acid formation and differential expression of tyrosine aminotransferase isoforms in *Anchusa officinalis* cell suspension cultures. Plant Cell Rep.

[CR49] Mizukami H, Ogawa T, Ohashi H, Ellis BE (1992). Induction of rosmarinic acid biosynthesis in *Lithospermum erythrorhizon*cell suspension cultures by yeast extract. Plant Cell Rep.

[CR50] Morrison TA, Kessler JR, Hatfield RD, Buxton DR (1994). Activity of two lignin biosynthesis enzymes during development of maize internode. J Sci Food Agric.

[CR51] Mousavi SM, Shabani L (2019). Rosmarinic acid accumulation in *Melissa officinalis* shoot cultures is mediated by ABA. Biol Plant.

[CR52] Ozturk M, Duru ME, Ince B, Harmandar M, Topcu G (2010). A new rapid spectrophotometric method to determine the rosmarinic acid level in plant extracts. Food Chem.

[CR53] Rakwal R, Tamogami Sh, Agrawal GK, Iwahashia H (2002). Octadecanoid signaling component ‘‘burst’’ in rice (*Oryza sativa* L.) seedling leaves upon wounding by cut and treatment with fungal elicitor chitosan. Biochem Biophys Res Commun.

[CR54] Reymond P, Farmer EE (1998). Jasmonate and salicylate as global signals for defense gene expression. Curr Opin Plant Biol.

[CR55] Ru M, Wang K, Bai Z, Peng L, He S, Wang Y, Liang Z (2017). A tyrosine aminotransferase involved in rosmarinic acid biosynthesis in *Prunella vulgaris* L. Sci Rep.

[CR56] Sathiyabama M, Bernsteinb N, Anusuya S (2016). Chitosan elicitation for increased curcumin production and stimulation of defence response in turmeric (*Curcuma longa* L.). Ind Crops Prod.

[CR57] Singleton V, Rossi JA (1965). Colorimetry of total phenolics with phosphor molybdic-phosphotungstic acid reagents. Am J Enol Viticult.

[CR58] Suzuki N, Miller G, Morales J, Shulaev V, Torres MA, Mittler R (2011). Respiratory burst oxidases: the engines of ROS signaling. Curr Opin Plant Biol.

[CR59] Trotel-Aziz P, Couderchet M, Vernet G, Aziz A (2006). Chitosan stimulates defense reactions in grapevine leaves and inhibits development of *Botrytis cinerea*. Eur J Plant Pathol.

[CR60] Wang CL, Liang ZS, Li DR, Yang JL (2011). Regulation of water-soluble phenolic acid biosynthesis in *Salvia miltiorrhiza* Bunge. Appl Biochem Biotechnol.

[CR61] Wanner LA, Li G, Ware D, Somssich IE, Davis KR (1995). The phenylalanine ammonia-lyase gene family in *Arabidopsis thaliana*. Plant Mol Biol.

[CR62] Wasternack C (2007). Jasmonates: an update on biosynthesis, signal transduction and action in plant stress response, growth and development. Ann Bot.

[CR63] Yan Q, Shi M, Ng J, Wu JY (2006). Elicitor-induced rosmarinic acid accumulation and secondary metabolism enzyme activities in *Salvia miltiorrhiza* hairy roots. Plant Sci.

[CR64] Zaho J, Sakai K (2003). Multiple signaling pathways mediate fungal elicitor-induced ß-thujaplicin biosynthesis in *Cupressus iusitanica* cell cultures. J Exp Bot.

[CR65] Zhang J, Simmons C, Yalpani N, Crane V, Wilkinson H, Kolomiets M (2005). Genomic analysis of the 12-oxophytodienoic acid reductase gene family of *Zea mays*. Plant Mol Biol.

[CR66] Zhang SH, Yan Y, Wang B, Liang Z, Liu Y, Liu F, Qi Zh (2014). Selective responses of enzymes in the two parallel pathways of rosmarinic acid biosynthetic pathway to elicitors in *Salvia miltiorrhiza* hairy root cultures. J Biosci Bioeng.

